# Influence of Self-Perceived Burden on Quality of Life in Patients with Urostomy Based on Structural Equation Model: The Mediating Effects of Resilience and Social Support

**DOI:** 10.1155/2022/9724751

**Published:** 2022-11-28

**Authors:** Yan Chang, Xiao Na Zhang, Fang Yu, Rui Zhang, Xin Dan Li, Jie Zhao, Hong Yan Lu

**Affiliations:** ^1^Department of Nursing, General Hospital of Ningxia Medical University, Yinchuan 750004, China; ^2^Nursing College of Ningxia Medical University, Yinchuan 750004, China

## Abstract

**Objective:**

To investigate the current situation of self-perceived burden in patients with urostomy, analyze the correlation between self-perceived burden and quality of life, and explore the intermediary role of resilience and social support.

**Methods:**

The convenience sampling method was used to select 303 patients with urostomy of outpatient departments of the three tertiary hospitals in Yinchuan, Ningxia region, China, from April 1, 2020, to October 1, 2020, who then completed a survey questionnaire. The survey questionnaire contained a general data questionnaire and self-perceived burden scale, city of hope-quality of life-ostomy questionnaire, Connor-Davidson resilience scale, and social support rating scale.

**Results:**

Self-perceived burden was present among 89.8% patients with urostomy; the quality of life of patients with urostomy is low. The results showed that the self-perceived burden and quality of life, resilience, and social support are related in pairs; self-perceived burden was significantly negatively correlated with quality of life，resilience, and social support; there was a significant positive correlation between quality of life, resilience, and social support; resilience and social support were parallel mediators.

**Conclusions:**

Patients with urostomy had a heavy self-perceived burden and low quality of life. Reducing the self-perceived burden of patients with urostomy by improving the level of resilience and social support, could raise the level of quality of life. This study could provide empirical basis for nurses to take continuous nursing intervention measures in order to reduce the self-perceived burden of patients with urostomy and ultimately to improve the quality of life.

## 1. Introduction

Studies have shown that each year, about 300 thousand new bladder cancer patients worldwide account for 3.2% of the new cancer cases [1]. The incidence rate increased with age, and the highest incidence was among 50~70 years old patients. In China, the incidence rate of bladder cancer ranks first in China's male urogenital malignancies and ranks eighth in the national malignant tumor [2]. In recent 10 years, the incidence rate of bladder cancer is increasing year by year [3]. The “golden standard” for bladder cancer treatment was radical total bladder resection combined with urinary diversion [4], after operation, urine flowed out through permanent abdominal stoma. First of all, the changes of body image and urination mode had changed the patient's original routine of life to a great extent, which had greatly troubled their physical and psychological aspects. Secondly, because the treatment of the disease and the management of urostomy had disrupted the normal social system and patients needed the long-term care and supports of family members, so patients with urostomy were very likely to have a self-perceived burden (SPB) [5], that was, patients believed that “I am a burden and a burden to others”. In the long-term development of SPB, patients would appear a series of negative emotions such as guilt, self-blame, anxiety, and reduced sense of self-worth. Studies had shown [6–8] that patients with urostomy had a difficult psychological experience after operation, and occurred a serious SPB, which seriously affected their quality of life (QOL). Previous studies on the factors affecting the quality of life of patients with urostomy mostly focused on demographic data and complications, while studies on the impact of social and psychological factors on the quality of life was few and not deep enough [9, 10]. Therefore, medical staff should pay more attention to the psychological health of patients while paying attention to the physiological function of urostomy. In recent years, the theory of positive psychology has gradually attracted extensive attention from domestic and foreign scholars. Resilience, which was a type of positive psychology, was considered to promote individual mental health, the prognosis of the disease, and patient's quality of life, and play a mediating role in the process [11–13]. Scholars believed that patients who are getting more social support and wider sources of information and having more communication with others were better at adopting positive coping styles, which could reduce the negative emotions of patients such as the self-perceived burden [14].

## 2. Methods

### 2.1. Subjects

From April 1, 2020, to October 1, 2020, patients with urostomy who met the inclusion and exclusion criteria in outpatient department of three tertiary hospitals in Yinchuan, Ningxia region, China were investigated. A total of 303 patients were included by convenience sampling method.

(1) Inclusion criteria were as follows: (1) bladder cancer was diagnosed by clinical and histopathological examination, and the operation way was “cystectomy + ileostomy or total cystectomy + ureterostomy”. (2) Clear consciousness, with normal communication and cognitive judgment ability. (3) Voluntarily filled in the questionnaire and signed the informed consent form.

(2) Exclusion criteria were as follows: (1) defected in important parts of the body; (2) there were dysfunction of heart, lung, kidney, and other organs, and other chronic diseases that seriously affect the quality of life, such as stroke and gout; (3) patients with tumor recurrence, metastasis, or other malignant tumors at the same time; (4) patients with severe mental illness

In the multifactor regression analysis, the sample size should be at least 5 ~ 10 times of the number of independent variables [15, 16]. In the study, SPBS had 3 dimensions, CD-RISC had 3 dimensions, C-COH had 4 dimensions, SSRS had 3 dimensions, patient general data had 20 variables, and the final total number of variables was 33. Considering the invalid questionnaire, on the original basis, 10% of the sample size had been expanded as the sample size of the survey, and the final calculated sample size was at least 182.

Zhou et al. [17] believed that the sample size for constructing the structural equation model should not be less than 200 cases, and it was better not to exceed 500 cases. The final sample size of this study was expanded to 300 cases.

### 2.2. Ethical Statement

This study was approved by the ethics review institution of the General Hospital of Ningxia Medical University (2020-643), complying with the declaration of Helsinki. Prior to data collection, consent and cooperation agreements were obtained from nine hospital administrators and departments, and prior to participation, all patients taking part in the study signed written informed consent forms.

### 2.3. Data Collection

When the patients visited the ostomy clinic, at the interval when they were queuing for treatment or at the end of treatment, we distributed and then collected questionnaires. The researcher explained the purpose and participation methods of this study to patients and family caregivers. We guaranteed that the information obtained would only be used for this study and would not disclose privacy. Followed the voluntary principle of patients, obtained the consent of caregivers, and then issued questionnaires after signing the informed consent form. Patients could quit without reason halfway through the questionnaire. If patients could not fill in the questionnaire personally, the researcher asked for the opinions of the patients one by one, and filled in the answers on behalf of the patients. All the questionnaires distributed were collected and checked on the spot. If any missing items were found, the subjects were asked to confirm and supplement them in time. A total of 307 questionnaires were distributed, of which 303 were valid, with an effective rate of 98.7%.

### 2.4. Research Tools

#### 2.4.1. General Information Questionnaire

The general information questionnaire consisted of 13 items, including age, gender, marital status, and education level. Urostomy operation time, stoma adaptation degree, stoma self-care degree, whether it is under treatment, whether there are complications, whether there are chronic diseases, main caregivers, health status of main caregivers, etc.

#### 2.4.2. Self-Perceived Burden Scale (SPBS)

SPBS was improved from 25 items to 10 items by Simmons [18], included three dimensions of physical factors, emotional factors, and economic factors. The scale translated and tested by Wu and Jiang [19] had good internal consistency, reliability, and validity, Cronbach's *α* score was 0.91. The SPBS score adopted a Likert 5 rating, from “never” (1 point) to “always” (5 points), with a total score that was either positive or negative (only the eighth item was scored in reverse, the others were positive scores). A higher total score indicates a higher level of individual SPB.

#### 2.4.3. City of Hope-Quality of Life-Ostomy Questionnaire (COH-QOL-OQ)

COH-QOL-OQ was originally constructed by Grant et al. [20]. In 2013, Gao and Yuan [21] translated and revised it into Chinese. It was verified to have good test characteristics among Chinese people, Cronbach's *α* score was 0.931. It had 32 items including four dimensions of mental health, physical health, mental health, and social health.

#### 2.4.4. Connor-Davidson Resilience Scale (CD-RISC)

The scale was compiled by American psychologist Connor and Davidson [22]. It was translated and revised into Chinese by Yu and Zhang [23]. CD-RISC consisted of three dimensions: tenacity, strength, and optimism, totaling 25 items. Cronbach's *α* score was 0.93 [24].

#### 2.4.5. Social Support Rating Scale (SSRS)

The author of SSRS is Chinese scholar, Xiao [25]. It had been verified to have good testing characteristics in Chinese population. At present, it has been widely used in various research fields. SSRS consisted of three dimensions: objective support, subjective support, and utilization support. The highest total score is 64 and the lowest is 12. The lower the total score, the worse the social support.

### 2.5. Statistical Analysis

We used the Excel to establish a database and SPSS 25.0 for statistical analysis and AMOS 21.0 software for constructing the structural equation model. The general information of patients with urostomy was analyzed by descriptive statistical analysis of frequency and percentage. The Pearson correlation analysis was performed to assess the relationship between SPB, quality of life, resilience, and social support.

## 3. Results

### 3.1. Participant General Information

Used the methods of one-way ANOVA and *t*-test to analyze the scores of self-perceived burden of patients with urostomy, it was found that the SPB scores were significantly different from 9 variables: age, work status, number of children, average monthly income, year of urostomy, adaptation to urostomy, stoma self-care ability, currently in treatment, and complications, *P* < 0.05. Specific data is shown in Table 1.

### 3.2. Scores of SPB, QOL, Resilience, and Social Support

The mean score of SPB was 31.00 (SD = 6.89; range 17-44), which reflected a moderate level of SPB among the patients with urostomy. The mean score of QOL was 194.57 (SD = 20.41), which reflected a low level of QOL among the patients with urostomy. The mean score of resilience was 51.22 (SD = 13.32), which reflected a relatively low level of resilience among the patients with urostomy. The mean score of social support was 29.62 (SD = 3.61), which reflected a moderate level of social support among the patients with urostomy. Means and standard deviation of all variables regarding SPB, resilience, QOL, and social support are shown in Table 2.

### 3.3. Correlations among SPB, QOL, Resilience, and Social Support

The results showed that the total SPB scores were negatively correlated with the total QOL, resilience, and social support scores. There was a positive correlation between QOL, resilience, and social support scores. There was a significant correlation between the SPB, QOL, resilience, and social support to each other. Specific results are shown in Table 2.

### 3.4. Construction of Structural Equation Model

#### 3.4.1. Construct the Initial Model of SEM and Modify the Model

**Figure 1:**
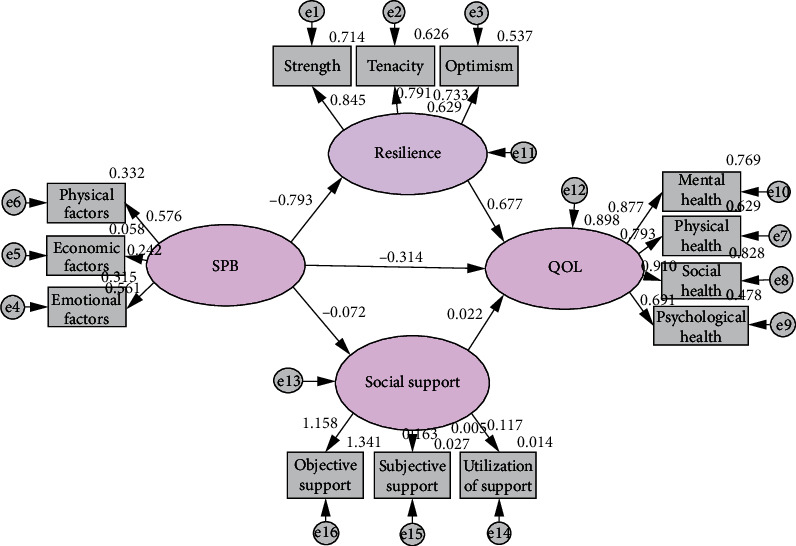
Structural equation model of parallel mediation of resilience and social support (Initial).

**Figure 2:**
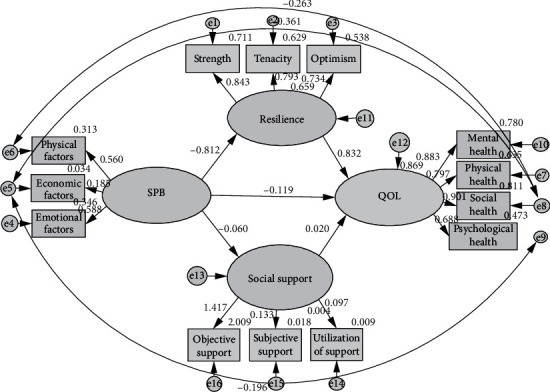
Structural equation model of parallel mediation of resilience and social support (Modified).

Because the initial model fit of SEM (Figure 1, Table 3) was poor and not ideal, so the model was modified according to modification index (MI). The results of each index of the modified model (Figure 2, Table 3) met the ideal excellent standard.

#### 3.4.2. The Mediating Effect of Resilience and Social Support in the Process of SPB Affecting QOL

The results of this SEM showed that the direct effect value was -0.119 and the indirect effect value was -0.676. The confidence interval did not include 0, indicating the existence of intermediary effect. It could be explained that resilience and social support played a parallel intermediary role in the impact path of SPB on QOL. The total effect of SPB on QOL was -0.795, and the mediating effect accounted for 85.03% of the total effect. Significance test results of mediating effect of SEM are shown in Table 4.

## 4. Discussion

In this study, 56.1% of the patients had stoma for 5 ~ 17 years. 89.8% of the patients had SPB of different degrees, 55.4% of them had moderate burden, and 9.9% of them had severe burden. It could be seen that SPB was common in patients with urostomy. The level of SPB of patients with urostomy was different in age. The older the age, the heavier the SPB. Studies in China and abroad, there were different conclusions on the relationship between the age of cancer patients and SPB. Simmons [18] pointed out that SPB had nothing to do with patients' age, while Vanorden et al. [26] showed that SPB had a positive correlation with age. This study found that older patients had higher SPB scores than younger patients. It might be because of the physical function and health level were gradually declining with the growth of age, and the probability of needing the care of family members was increased. With other diseases such as urostomy, they would be more depending on family members, which would aggravate the physical and mental pain and increase the SPB [27].

It could be seen from the results of this study that the SPB score of nonworking, sick, or retired patients with urostomy was higher than that of part-time workers and more than that of full-time workers, similar to the results that Zhang et al. [28] and Wu and Jiang [19] reported. It might be related to the reduction of income without work, which would increase the economic pressure. Work symbolized income and determined social status, which could further prove that economic income was one of the most important factors affecting the level of SPB [29]. It might also be that working outside could be integrated into different social relations, which was conducive to dispersing the negative emotions caused by diseases and realizing the value of life. Therefore, the SPB of people working full-time was relatively light.

The results showed that the SPB score of patients who did not adapt to urostomy was significantly higher than that of patients who was perfectly adapted. The SPB of the patients being treated was also heavier, similar to the results that Xu et al. reported [30]. It might be due to the fact that the treatment not only increased the economic burden and physical pain but also aggravated the psychological burden due to the trouble caused by repeated hospitalization and on the way to medical treatment. Therefore, medical staff should pay attention to a patient's overall health status as well as disease status to reduce the risk of SPB.

In this study, the independent variable was SPB; the dependent variable was QOL. Resilience and social support were intermediate variables. A structural equation model was established to analyze and verify the causal relationship between the four. The results of this model showed that SPB had a direct negative effect on QOL, and could also indirectly affect QOL through the two parallel mediating variables: resilience and social support. At the same time, resilience and social support had a direct positive effect on QOL. As an intermediary variable, resilience and social support indirectly affected the relationship between SPB and QOL, playing a significant dual intermediary role. The intermediary effect accounted for 85.03% of the total effect. The results showed that SPB could not only directly affect the QOL of patients with urostomy but also indirectly affected the mental health status of patients through the intermediary effecting of resilience and social support, and finally affected the QOL of patients. Compared with previous studies [31, 32], our study has several distinct differences. Frist, the SPB of patients with urostomy caused them having negative emotions such as self-accusation, guilt, and self-depreciation for a long time. Such negative emotions made the patients unable to face the harm caused by bladder cancer correctly. Second, it influenced the treatment and nursing, and was not conducive to the improvement of the quality of life. In the process of adaptation and acceptance of urostomy patients, resilience and social support played a positive role in the stage of patients' self-psychological adjustment, which was the source of power to realize self-psychological transformation [33]. As a protective factor of individual mental health, resilience had been confirmed as an intermediary variable in many studies [34–36]. Third, this study also further verified that resilience played an effective intermediary effect. This study showed that social support played an important guiding role in patients' physical and mental health behavior. Therefore, nurses could take resilience as the focus of nursing intervention measures. Through effective psychological nursing for patients, such as cognitive behavior therapy, mindfulness therapy, and music therapy, nurses could reduce the impact of their SPB on the quality of life of patients with urostomy by improving the level of resilience. Strengthen the social support for urostomy patients, so as to reduce their SPB, and in order to improve their quality of life.

## 5. Conclusion

Patients with urostomy had a heavy self-perceived burden and low quality of life. The SPB of patients with urostomy would eventually affect the quality of life, in which resilience and social support played a parallel intermediary role. Therefore, nurses could strengthen continuous nursing by taking personalized psychological intervention measures, to help patients to adjust cognition, emotion, and psychology. Through improving the level of resilience and social support to reduce the SPB of patients with urostomy, so as to finally improve the patients' quality of life.

This study was a cross-sectional survey in the form of questionnaire, and the research method was relatively simple. Due to the actual conditions and time constraints, only the three tertiary hospitals in Yinchuan were included, and the representativeness of the samples might be affected. The research results needed to be further verified and promoted.

## Figures and Tables

**Table 1:** Participants' characteristics and differences of the SPB in different groups (*N* = 303).

Characteristic	*N* (%)	−*x* ± *s*	*t*/*F*	*P*
Age			4.520^∗^	0.001
30~	6 (2.0)	26.50 ± 8.07		
40~	40 (13.2)	28.90 ± 5.06		
50~	79 (26.1)	30.03 ± 7.29		
60~	112 (37.0)	31.16 ± 7.08		
70~	66 (21.8)	33.59 ± 6.17		
Work status			3.224^∗^	0.023
On job	45 (14.9)	28.73 ± 6.14		
Part time work	68 (22.4)	30.15 ± 7.22		
Sick leave/retire	101 (33.3)	31.47 ± 6.94		
No job	89 (29.4)	32.28 ± 6.66		
Number of children			3.423^∗^	0.009
0	4 (1.3)	34.25 ± 4.50		
1	108 (35.6)	29.19 ± 6.43		
2	120 (39.6)	31.59 ± 7.00		
3	53 (17.5)	32.30 ± 7.04		
4 or more	18 (5.9)	33.39 ± 6.83		
Income (RMB)/month			22.253^∗^	0.001
<1000	62 (20.5)	35.18 ± 6.08		
1000~	131 (43.2)	31.72 ± 6.47		
3000~	96 (31.7)	28.47 ± 6.15		
5000~	14 (4.6)	23.21 ± 5.60		
Year of urostomy			15.169^∗^	0.001
≤1	28 (9.2)	33.25 ± 5.50		
2 ~ 4	105 (34.7)	34.07 ± 6.78		
5 ~ 7	81 (26.7)	30.40 ± 6.50		
8 ~ 10	46 (15.2)	27.09 ± 5.85		
≥11	43 (14.2)	27.40 ± 5.75		
Adaptation to urostomy			17.441^∗^	0.001
Fully adapted	251 (82.8)	30.02 ± 6.85		
Partial adaptation	49 (16.2)	35.45 ± 4.77		
Unable to adapt	3 (1.0)	40.67 ± 1.53		
Stoma self-care ability			42.787^∗^	0.001
Completely self-care	184 (60.7)	28.42 ± 6.21		
Need help	93 (30.7)	34.67 ± 6.29		
Rely entirely on others	26 (8.6)	36.19 ± 4.20		
Currently in treatment			3.076	0.002
Yes	18 (5.9)	35.78 ± 5.95		
No	285 (94.1)	30.70 ± 6.84		
Complications			-8.47	0.001
No	213 (70.3)	29.04 ± 6.48		
Yes	90 (29.7)	35.64 ± 5.46		

^∗^: F; *P* < 0.05.

**Table 2:** Correlations among SPB, resilience, QOL, and social support (*N* = 303).

Variable	−*x* ± *s*	Range	1	2	3	4
SPB	31.00 ± 6.89	17~44	1			
Resilience	51.22 ± 13.32	28~86	-0.896^∗∗^	1		
QOL	194.57 ± 20.41	142~234	-0.905^∗∗^	0.831^∗∗^	1	
Social support	29.62 ± 3.61	17~40	-0.144^∗^	0.140^∗^	0.127^∗^	1

^∗∗^ *P* < 0.01: statistically significant. ^∗^*P* < 0.05: statistically significant.

**Table 3:** Fitting index of SEM of patients with urostomy.

Project	*x* ^2^/df	RMSEA	CFI	AGFI	TLI	NFI	GFI	IFI
Reference	<2	<0.05	>0.95	>0.90	>0.95	>0.90	>0.95	>0.95
Initial model	2.280	0.065	0.953	0.898	0.940	0.921	0.932	0.954
Final model	1.596	0.044	0.979	0.926	0.972	0.947	0.953	0.980

**Table 4:** Significance test of mediating effect in structural equation model.

Effects	Standardized regression weights		*P*	Bootstrap confidence (BC)	
	Estimate	SE		Lower bounds	Upper bounds
Direct effects	-0.119	0.241	0.451	-0.433	-0.331
Indirect effects	-0.676	0.227	0.002	-1.362	-0.485
Total effects	-0.795	0.062	0.001	-0.932	-0.680

## Data Availability

The data used to support the findings of this study are available from the corresponding author upon request.

## Supplementary Materials

Supplementary Materials This study was approved by the ethics review institution of The General Hospital of Ningxia Medical University (2020-643) (Supplementary Materials).
