# Supplementary material for: Influence of Self-Perceived Burden on Quality of Life in Patients with Urostomy Based on Structural Equation Model: The Mediating Effects of Resilience and Social Support
Source: Biomed Res Int. 2022 Nov 28;2022:9724751. doi: 10.1155/2022/9724751 (PMC9722293; doi:10.1155/2022/9724751)
Supplement: Supplementary Materials — This study was approved by the ethics review institution of The General Hospital of Ningxia Medical University (2020-643) (Supplementary Materials). [file 9724751.f1.pdf]

Ethical Clearance Certificate of Ningxia Medical University General Hospital Scientific Research Ethics Committee

**Project name:**	Study on Correlation among the Self-perceived Burden, Quality of Life and Resilience in Patients with Urostomy	**Ethics number:**	2020-643
**Project leader:**	Lu Hongyan	**Professional titles:**	chief nurse
**Phone number:**	13619504803	**Department:**	Nursing Department

Research unit responsible for the project: Ningxia Medical University General Hospital

Cooperative research unit:
None

Date: **From** 2020-04-01 **To** 2021-06-30

Sources for research projects:
Autonomy

Longitudinal Study（Government support）
☐ **Association / Foundation** ☐ **Company** ☐ **Horizontal cooperation** ☐ **Multicenter Study** ☑ **Autonomy** ☐ **Others**

Research funding unit:
Other

Subjects: ☐ **Animals** ☐ **Cells** ☐ **Imaging Data** ☑ **Human Specimens and Samples**

Reviewing opinions :

☐ **This study comply with ethical requirements, and can be tested according to this scheme.**

☐ **This study does not comply with ethical requirements. Please revise the application before ethics committee review it again.**

Do you have patient consent form? ○ **Yes** ○ **No**

Whether the methods of obtaining patient consent were appropriate: ○ **Inappropriate** ○ **Appropriate**

Ningxia Medical University General Hospital
Scientific Research Ethics Committee

agree!

Wei Zhao   Yanrong Wang   Bao zhen Yang

XiaoYun Ding   Fanqin Yang

Hongwan Dang   Yu Lin Guo
